# Aberrant high expression of immunoglobulin G in epithelial stem/progenitor-like cells contributes to tumor initiation and metastasis

**DOI:** 10.18632/oncotarget.5542

**Published:** 2015-10-12

**Authors:** Qinyuan Liao, Wei Liu, Yang Liu, Fulin Wang, Chong Wang, Jingxuan Zhang, Ming Chu, Dongyang Jiang, Lin Xiao, Wenwei Shao, Zhengzuo Sheng, Xia Tao, Lei Huo, C. Cameron Yin, Youhui Zhang, Gregory Lee, Jing Huang, Zihai Li, Xiaoyan Qiu

**Affiliations:** ^1^ Department of Immunology, School of Basic Medical Sciences, Peking University, Beijing, 100191, China; ^2^ Peking University Center for Human Disease Genomics, Beijing, 100191, China; ^3^ Key Laboratory of Medical Immunology, Ministry of Health, Beijing, 100191, China; ^4^ Department of Pathology, Chinese PLA General Hospital, Beijing, 100853, China; ^5^ Department of Gynecology, Peking University First Hospital, Beijing, 100034, China; ^6^ Division of Pathology and Laboratory Medicine, The University of Texas MD Anderson Cancer Center, Houston, Texas, 77030, USA; ^7^ Department of Hematopathology, The University of Texas MD Anderson Cancer Center, Houston, Texas, 77030, USA; ^8^ Department of Immunology, Cancer Institute & Hospital, Chinese Academy of Medical Science, Beijing, 100021, China; ^9^ Andrology Lab, University of British Columbia Centre for Reproductive Health, Vancouver, BC V5Z 4H4, Canada; ^10^ Department of Microbiology and Immunology, Medical University of South Carolina, Charleston, SC 29425, USA

**Keywords:** IgG, RP215, epithelial stem/progenitor-like cells, tumor metastasis

## Abstract

High expression of immunoglobulin G (IgG) in many non-B cell malignancies and its non-conventional roles in promoting proliferation and survival of cancer cells have been demonstrated. However, the precise function of non-B IgG remains incompletely understood. Here we define the antigen specificity of RP215, a monoclonal antibody that specifically recognizes the IgG in cancer cells. Using RP215, our study shows that IgG is overexpressed in cancer cells of epithelial lineage, especially cells with cancer stem/progenitor cell-like features. The RP215-recognized IgG is primarily localized on the cell surface, particularly lamellipodia-like structures. Cells with high IgG display higher migration, increased invasiveness and metastasis, and enhanced self-renewal and tumorgenecity ability *in vitro* and *in vivo*. Importantly, depletion of IgG in breast cancer leads to reduced adhesion, invasion and self-renewal and increased apoptosis of cancer cells. We conclude that high expression of IgG is a novel biomarker of tumor progression, metastasis and cancer stem cell maintenance and demonstrate the potential therapeutic benefits of RP215-recognized IgG targeted strategy.

## INTRODUCTION

Immunoglobulin (Ig) genes were evolved approximately 500 million years ago and exist in nearly all classes of jawed vertebrates. To date, almost all of the significant findings in the field have centered on B cell-derived Ig (B-Ig). We have first reported that a variety of non-B cells, such as breast, liver and lung cancer cells, but not their normal counterpart, surprisingly express high level of IgG [1]. Our following studies demonstrated that the IgG expressed in cancer cells has the ability to promote tumor cell growth [2]. Subsequently, our and other groups have found that many forms of Igs, including IgG, IgA, and IgM (known as non-B-Igs) are widely expressed in non-B-cells of different origins, especially cancer cells [3–13].

To recognize a variety of antigens, the Ig gene is rearranged to yield a vast repertoire of antigen receptor-binding specificities during B cell development. This process involves two stages of rearrangement, with the first being the assembly of the variable (V), diversity (D), and joining (J) gene segments of the heavy chain (IgH) and the V and J gene segments of the light chain (IgK). Rearrangement enables B-Igs to display a great diversity in an individual. We have analyzed sequences of thousands of non-B-Igs genes from isolated epithelial cells, neuron, and spermatogenic cells in human and mouse [14–17]. Clearly, all of non-B-Igs also display classical V_H_DJ_H_ and VκJκ recombination, however, comparing to B-Igs, the non-B-Igs display several unique genetic characteristics. Firstly, cells of the same linage from an individual, even from different individuals, frequently use several conserved sets of V_H_DJ_H_ and VκJκ rearrangements [14, 15]. Secondly, the V_H_DJ_H_ rearrangements of different classes of IgH, such as gamma chain of IgG and mu chain of IgM, in the same cell show their own special V_H_DJ_H_ pattern [14]. These indicate that a non-canonical mechanism of class switch recombination is present in non-B cells, and each class of Ig in the same cell may exert their own specific functions. Thirdly, V_H_ in cancer cells usually exhibits hypermutation, while V_H_ region keeps the germline sequence in normal cells [16], suggesting that the V_H_ hypermutation may be involved in tumorgenesis.

Previous studies from our and other groups demonstrated that IgG is prominently overexpressed in many cancers [2–12], but expressed at a very low level in their normal counterparts. In addition, IgH gene copy number is significantly increased in rat breast cancer induced by chemical carcinogen [21]. Furthermore, IgG expression level is positively correlated with low differentiation of cancer cells [18, 19]. Importantly, cancer cell-derived IgG promotes cancer cell proliferation and survival *in vitro* and *in vivo* [2, 20]. Yet, the precise effect of non-B-IgG in cancer initiation and progression remain elusive.

RP215, a monoclonal Ab, react with ovarian cancer cells using the extract of the ovarian cancer cell line OC-3-VGH as an immunogen [21]. It was shown that RP215 also reacts with human cancer cells of many other tissue origins but does not react with cells from normal tissues [22]. The molecule recognized by RP215 is known as CA215 (cancer antigen 215) and has been considered as a pan cancer marker. CA215 is later identified as IgG, and sialic acid has been reported to be enriched in the RP215-affinity purified IgG [23, 24]. Moreover, RP215 is able to induce extensive apoptosis and significantly inhibit tumor growth *in vivo* [25, 26]. Taken together, we decided to explore the function of cancer-derived IgG using RP215 as a tool.

In this study, we identify that RP215 recognized IgG is prominently expressed in cancer cells of epithelial lineage, especially those with stem/progenitor-like cancer cell features. RP215 recognized IgG is involved in tumor initiation and progression by maintaining cancer stem cell features and promoting metastasis.

## RESULTS

### RP215 specifically recognizes IgG

**Figure 1:**
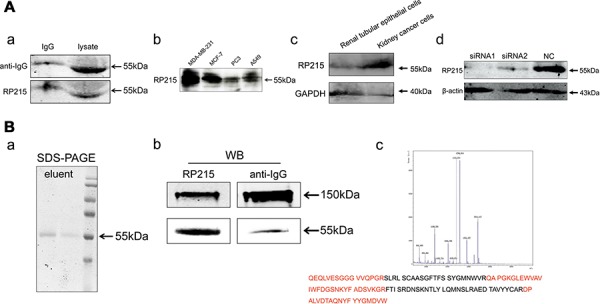
IgG is recognized by RP215 **A.** (a) The IgG was detected in purified human IgG (left, IgG, as positive control) and in cancer cell extracts of EpCAM-positive cancer cells isolated from ascitic fluid from patients with ovarian cancer (right, lysate) by Western blot using RP215, the commercialized anti-human IgG antibody as a control; (b) RP215 recognized IgG was detected in the cell lysate of several cancer cell lines respectively (MDA-MB-231, MCF-7, PC3 and A549); (c) IgG recognized by RP215 was detected in kidney cancer cells isolated from patient tissues and normal renal tubular epithelial cells from tumor adjacent of renal tissues; (d) IgG expression was determined after treatment with two siRNAs targeting the IgG heavy chain by Western blot analysis. NC as control siRNA. GAPDH was used as an internal control. **B.** By RP215-affinity chromatography, IgG in EpCAM^+^ cancer cells isolated from ascitic fluid was purified and analyzed by SDS-PAGE, Western blot and MS analysis. (a) The protein purified by RP215-affinity chromatography showed 55kDa, lane 1: the first tube of elution, lane 2: the last tube of elution, by SDS-PAGE; (b) the protein was determined by Western blot under non-reduced (150kDa) or reduced SDS-PAGE condition; (c) The 55kDa band was identified by MS analysis, and the peptide sequences high homologous to the VH3 family of Ig heavy chain are marked in red.

To identify the specificity of RP215 antibody, Western blot, affinity chromatography and mass spectrometry (MS) were performed using the whole cell lysate containing all cancer cell proteins. We determine that RP215 recognizes a single band of IgG heavy chain in cancer cell extracts from EpCAM (epithelial cell adhesion molecule)-positive cancer cells isolated from ascitic fluid of ovarian cancer patients, as well as several cancer cell lines, including breast cancer (MDA-MB-231 and MCF-7), prostate cancer (PC3) and lung cancer (A549) (Figure 1A a and 1A b). Moreover, we found that the IgG recognized by RP215 was high expressed in kidney cancer cells isolated from patient tissues, but few in the normal renal tubular epithelial cells from tumor adjacent of renal tissues (Figure 1A c). Knockdown of IgG heavy chain by RNA interference results in a reduction of IgG heavy chain band recognized by RP215 (Figure 1A d). Additionally, only IgG, but not other proteins in cancer cells, is affinity-purified by RP215 shown by SDS-PAGE, Western blot and mass spectrometry (Figure 1B). To address if the IgG recognized by RP215 has some unique patterns, we analyzed the VDJ pattern in several cancer cell lines, including MDA-MB-231, MCF-7 and SK-MES-1 (lung squamous cell carcinoma), recognized by RP215. The sequencing analyses show that each cancer cell line-derived IgG heavy chain has its own VDJ pattern, such as VH3-7/DH3-3/JH5 in MDA-MB-231, VH4-4/DH2-21/JH4 in MCF-7 and VH4-59/DH2-15/JH4 in SK-MES-1, suggesting that RP215 recognition is unrelated to any unique VDJ patterns and that the specific epitope recognized by RP215 should be a common epitope of cancer-IgG heavy chains.

### Overexpression of RP215-recognized IgG in epithelial cancer cells, but not in B lymphoid or mesenchymal originated cancer cells

**Figure 2:**
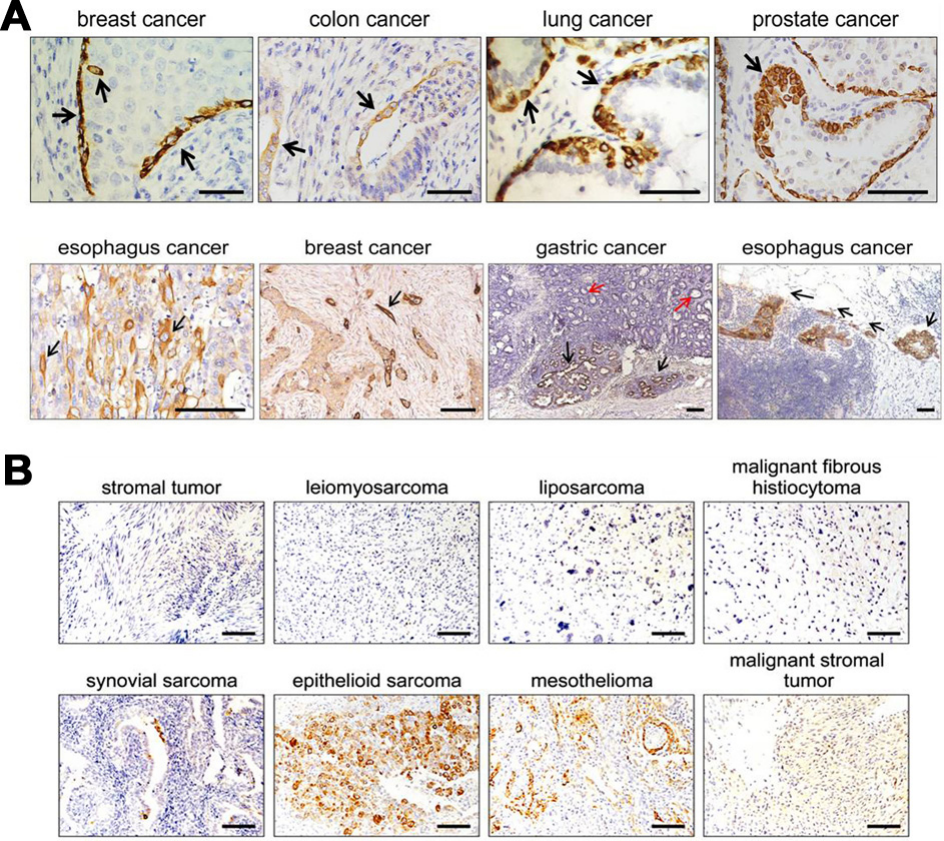
IgG recognized by RP215 was mainly limited to be expressed in some epithelial lineage of cells, especially stem cell-like cells **A.** RP215 expressed in human epithelial cancer tissues. Strong immunostaining was showed in a small population of basal/myoepithelial-like cancer cells of breast, colon, lung, and prostate (black arrows). And the cancer cells at the front of invasive tumors in esophagus cancer, breast cancer and gastric cancer (black arrows). Red arrows indicated for the cancer cells in the gastric cancer *in situ*. Scale bars, 100 μm. **B.** No immunostaining was observed in lymphomas or mesenchymal tumors, except for some cells with epithelial feature in synovial sarcoma, epithelioid sarcoma and mesothelioma. Scale bars, 50 μm.

RP215 recognizes many epithelial cancer cells [22], but much less is known about the detail expression profile of RP215-recognized epitope. we analyzed expression profile and distribution of RP215-recognized IgG in malignant cells, including epithelial, lymphoid and mesenchymal tissues. The immunohistochemical results revealed that the IgG is shown in almost all epithelial cancers including breast, prostate, colon, lung, gastric, ovarian and esophageal carcinomas. Unexpectedly, a low concentration of RP215 gave rise to a significant RP215 staining (IgG^high^) in a small population of basal/myoepithelial (which are considered to be adult stem/progenitor cells)-like cells [27], in the interior spaces of carcinoma cell layers, or in some invasive single or multiple clusters between 4–10 cancer cells (Figure 2A). Suggest that expression of IgG recognized by RP215 might be related to cancer cell regeneration, migration and invasion. No staining has been observed in lymphoid or mesenchymal originated tissues with the exception of a population of epithelial-like cancer cells in synovial sarcoma or epithelioid sarcoma (Figure 2B, Supplementary Table S1). These results suggest that RP215 mainly recognizes epithelial cancer cells, especially the epithelial CSC-like cells, but not those cells of non-epithelial origin.

Subsequently, we first analyzed expression profile of RP215-recognized IgG in normal tissues by immunohistochemical staining using RP215. The results show that RP215 is unable to react with normal tissues of multiple origins such as liver, kidney, lung, cervix, pancreas and endometria, brain, lymphocytes and mesenchymal cells. Only a small number of epithelial cells showed positive staining in the stomach, small intestine, thymus, and bile duct. Exceptionally, very strong RP215 staining is displayed in squamous cells, contained in the skin, esophagus and cervix, and some basal layer/myoepithelial cells, located in the breast glands, prostatic glands, bronchial epithelia and bronchial glands. No staining was seen with the luminal cells (Supplementary Figure S1). The unique immunostaining pattern of RP215 did not report in previous studies.

### IgG^high^ cells co-localize with CSCs as well as adult stem/progenitor cells of epithelial origins

**Figure 3:**
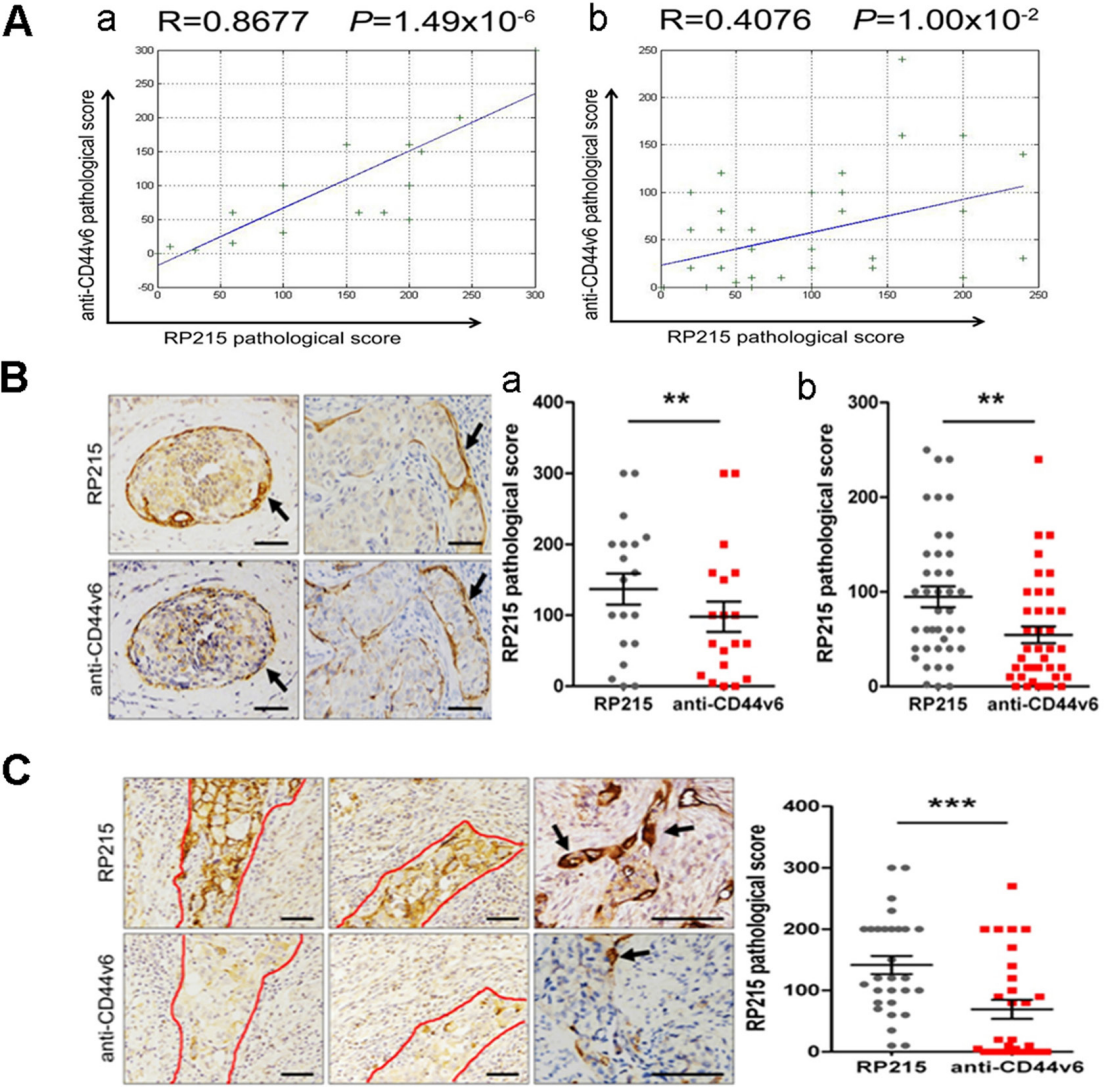
RP215^high^ cells displayed co-localization with CSCs **A.** RP215^high^ cells was co-expressed with CD44v6. The results indicated the correlation between RP215^high^ cells and CD44v6^+^ cells in infiltrating ductal carcinoma (a), and in ductal carcinoma *in situ* (b). R, Pearson correlation coefficient. **B.** The relationship between RP215^high^ cells and CD44v6^+^ cells in basal-like cells in breast cancer tissues was analyzed by immunohistochemistry (black arrows). Scale bars, 50 μm. Comparing the pathological scores between RP215^high^ cells and CD44v6^+^ cells in infiltrating ductal carcinoma (a) and in ductal carcinoma *in situ* (b). **C.** The relationship between RP215^high^ cells and CD44v6^+^ cells in invasive cancer cells in breast cancer tissues was analyzed by immunohistochemistry. Scale bars, 50 μm. Strong immunostaining of RP215 was shown in a small population of CSC-like cancer cells and some frontier cancer cells in breast cancer tissues (demarcated by red line and black arrows), more specific staining for the invasive cancer cells was showed by RP215 than that by anti-CD44, the significant differences are displayed in the column (on the right).

The CD44^+^/CD24^−/low^ cells are a subset of breast cancer stem cells (BCSCs) [28–30]. We first used the breast cancer as a model to analyze the correlation between IgG^high^ cells and CD44^+^/CD24^−/low^ BCSCs on adjoined sections by immunohistochemistry using RP215 and anti-human CD44v6, a CD44 isoform, which is more correlate to tumor invasion [31]. Our results clearly indicated a strong positive correlation and co-localization between RP215^high^ cells and CD44v6^+^ cells in the basal-like or invasive cancer cells (correlation coefficient R = 0.6794, *P* = 4.53 × 10^−9^, *n* = 58), but no correlation or co-localization was found between IgG^high^ cells and CD24^+^ cells (Supplementary Figure S2A). Importantly, a stronger correlation has been found between IgG^high^ cells and CD44v6^+^ cells in infiltrating ductal carcinoma (R = 0.8677, *P* = 1.49 × 10^−6^, *n* = 19) than in ductal carcinoma *in situ* (R = 0.4076, *P* = 1.00 × 10^−2^, *n* = 39) (Figure 3A and 3B). Furthermore, these basal-like cells, invasive cancer cells and cells at the leading edge of cancer show a higher frequency of RP215 staining than those of anti-CD44v6 staining (Figure 3C). Suggest that RP215-recognized IgG may be more correlation with BCSCs than that of CD44V6. Both CD133 and ALDH1 are also considered as BCSC markers, which are considered to be different subpopulation of BCSC from CD44^+^/CD24^−^ BCSCs [32–33]. We also analyzed correlations between RP215^high^ cells and CD133 and ALDH1. No significant correlation was found between either IgG^high^ cells and CD133 or IgG^high^ cells and ALDH1 (Supplementary Figure S2B). These observations suggest that RP215 mainly recognizes the subpopulation of CD44^+^/CD24^−^ BCSCs.

In general, tumor initial cells (TICs)/cancer stem cells (CSCs) display similar characteristics and markers as their normal lineage-restricted stem/progenitor cells. p63, which is a well-accepted lineage-restricted stem/progenitor cell marker of stratified epithelium [34] As expected, our results show that strong RP215 staining in squamous cells (which is described to have the properties of restricted stem/progenitor cells), and in basal/myoepithelial cells of stratified epithelium, and co-localizes with p63. However, almost no staining was found in differentiated epithelial cells (Supplementary Figure S2C). Indicate that RP215-recognized IgG is also overexpressed in adult stem/progenitor cell of stratified epithelium, but not the differentiated cells.

### Increasing RP215-recognized cells in breast tissue predicted metastasis and correlate with poor prognosis factors

**Figure 4:**
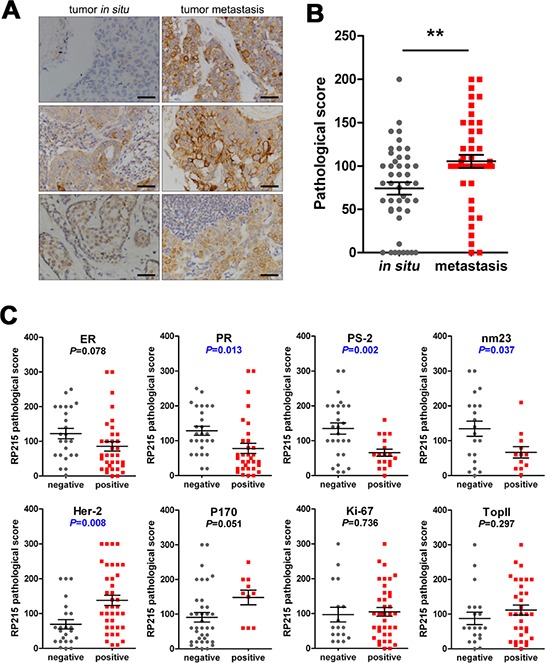
Expression frequency of RP215-positive cells was significantly high in metastatic cancers, which predicted metastasis and poor prognosis **A.** and **B.** The RP215 expression level in 45 matching pairs of breast cancer tissues and their corresponding lymph node metastases was analyzed by immunohistochemistry. Scale bars, 50 μm. Error bars show means ± standard error. **P < 0.005 by the Student *t*-test. **C.** The correlation between RP215 and several well-accepted prognostic indicators of breast cancer. Strong RP215 staining was negatively correlated with the expression of PR (*P* = 0.013), PS-2 (*P* = 0.002), and nm23 (*P* = 0.037), while positively correlated with Her-2 (*P* = 0.008) and P170 (*P* = 0.051). RP215 staining did not correlate with Ki-67 (*P* = 0.736) or topoisomerase II (*P* = 0.297). **P < 0.005 by the Student *t*-test.

We first analyzed the significance of RP215 reactivity in the evaluation of cancer metastasis using a TMA including 45 pairs of breast cancer and their lymph node metastases. We found that much stronger staining was observed in the metastatic cancer cells than *in situ* breast cancer (Figure 4A and 4B). Furthermore, using 107 cases of breast cancer, we analyzed the significance of RP215 staining in clinical prognosis as compared with several well-accepted prognostic indicators. The estrogen receptor (ER), progesterone receptor (PR), PS-2 (an regulated protein of estrogen) and nm23 (a tumor metastasis inhibitory gene) have been considered good prognostic indicators in breast cancer [35–37]. as well as human epidermal growth factor receptor 2 (Her-2), an independent prognostic factor for poor prognosis of breast cancer, and P-glycoprotein (P170) have been associated with poor prognosis [38] Our results clearly showed that strong RP215 staining was negatively correlated with the expression of PR (*P* = 0.013), PS-2 (*P* = 0.002), and nm23 (*P* = 0.037). As expected, RP215 staining was positively correlated with Her-2 *(P* = 0.008) and P170 (*P* = 0.051). However, RP215 staining did not correlate with the expression of Ki-67, a marker of cell proliferation (*P* = 0.736), or topoisomerase II, another indicator of poor prognosis [39] (*P* = 0.297) (Figure 4C).

### IgG^high^ cells displayed high migration, metastasis and CSCs-like characteristics

**Figure 5:**
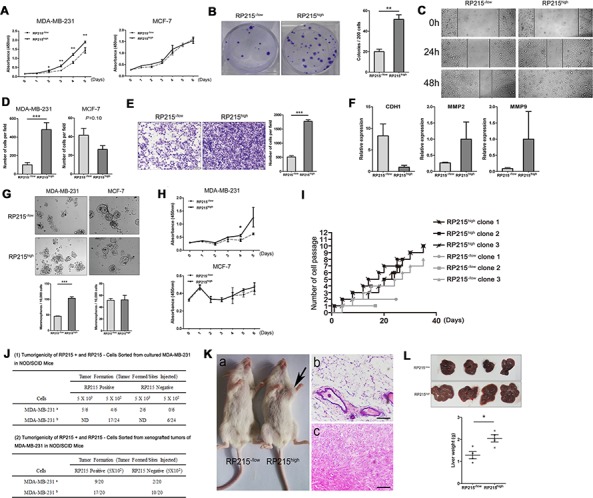
To confirm the involvement of RP215^high^ in the properties of CSCs in sorted RP215^high^ MDA-MB-231 or MCF-7 Cell, the proliferation, division, migration, invasion capacity, as well as the mammospheres formation, drug resistance to chemotherapy drugs, and the tumor initiation and metastasis capacity were determined *in vivo*, compared with the RP215-/low cells **A.** and **B.** Proliferation of RP215^high^ MDA-MB-231 cells was detected by CCK8 and clone formation assay. **C.** The migration ability of RP215^high^ MDA-MB-231 cells was analyzed by wound-healing. Images were taken at 0, 24, and 48 h. **D.** The migration ability of RP215^high^ MDA-MB-231 or MCF-7 cells was analyzed by Transwell assay. Data were taken 24 h in MDA-MB-231, and 96 h in MCF-7. **E.** The invasion capacity of RP215^high^ MDA-MB-231 cells was detected in Transwell inserts coated with matrigel matrix. Images were taken at 48 h. **F.** Expression level of CDH1, MMP-2 and MMP-9 was determined in RP215^high^ MDA-MB-231 cells by qRT-PCR. **G.** Mammospheres were generated from 10,000 RP215^high^ MDA-MB-231 or MCF-7 cells sorted by FACS in suspension culture, and then counted. **H.** Drug resistance capacity of RP215^high^ MDA-MB-231 or MCF-7 cells to paclitaxel was analyzed by CCK8 assay. **I.** Division ability of RP215^high^ MDA-MB-231 single cell was analyzed by continuous cell passage for 35 days. **J.** The number of outgrowths generated in NOD/SCID mice fat pads by RP215^−/low^ and RP215^high^ MDA-MB-231 cells. *a*, for the 1^st^ experiment; *b*, for the 2^nd^ experiment. **K.** a, representative tumor grown in NOD/SCID mice at the RP215^high^ cells' injection site. No tumor was detected at the RP215^−/low^ cells' injection site. b & c, H & E staining of RP215^high^ cells' injection site, revealing presence of tumor cells (c), the RP215^−/low^ cells' injection site contained only mouse tissue (b). Scale bars, 50 μm. **L.** The metastatic potential of sorted RP215^−/low^ and RP215^high^ MDA-MB-231 cells to liver was observed by spontaneous metastasis assays *in vivo*. RP215^−/low^ and RP215^high^ MDA-MB-231 cells (1 × 10^6^) were subcutaneously injected into the right mammary fat pad of 6-week-old female BALB/c nude mice. After 6 weeks, the mice were sacrificed and dissected. And the metastatic tumors in liver were pathologically examined, and these livers were weighted and data was analyzed. Error bars show means ± standard errors from individual experiments with three replicate assays. *P < 0.05; ** *P* < 0.01; ***P < 0.001 by the Student *t*-test.

To further confirm the involvement of RP215-recognized IgG in the properties of CSCs, we have analyzed the frequency of RP215 staining on cell surface between MDA-MB-231 (a claudin-low breast cancer cell-line, which was described for their properties of CSCs) and MCF-7 (a luminal-like breast cancer cell-line, which was described for their limited abilities to proliferate and migrate) [40–41] by fluorescence-activated cell sorting (FACS). It was found that higher percentage of RP215-recognized cells was found in MDA-MB-231 than in MCF-7. Next, the IgG^high^ and IgG^−/low^ cells of MDA-MB-231 and MCF-7 cells were sorted by FACS (Supplementary Figure S3). Initially, in MDA-MB-231, but not in MCF-7, we found that IgG^high^ cells displayed a greater ability to proliferate, migrate and invade as compared with IgG^−/low^ cells (Figure 5A–5E) by CCK8, clone formation, wound healing, Transwell, and transwell inserts coated with matrigel matrix assay. Moreover, the expression of E-cadherin (CDH1), a negative-related protein for tumor invasion, was clearly reduced, and two positive-related proteins for tumor invasion and metastasis, MMP-2 and MMP-9 [42], were increased in expression in the IgG^high^ MDA-MB-231 cells (Figure 5F).

To further analyze if IgG^high^ cells have CSC-like properties, we first performed sphere formation and drug-resistance assays to determine their self-renewal and drug-resistance abilities *in vitro*. IgG^high^ MDA-MB-231 cells displayed significantly higher sphere-forming efficiency and resistance to paclitaxel than that of IgG^−/low^ cells. However, no obvious difference in these properties was observed when comparing IgG^high^ and IgG^−/low^ MCF-7 cells (Figure 5G and 5H). In addition, CSC has a great division potential. Subsequently, we have compared clone formation and division potential between IgG^high^ and IgG^−/low^ MDA-MB-231 cells dependent on the single cell clone formation assay, it was found that both IgG^high^ cell and IgG^−/low^ cell could come into being cell clones, however, IgG^high^ cell clones show a rapid growth ability than IgG^−/low^ cells. To compare the division potential between IgG^high^ and IgG^−/low^ MDA-MB-231 clones. We selected 3 independent IgG^high^ and 3 independent IgG^−/low^ MDA-MB-231 clones, and continuously culture *in vitro* for 36 days. Observably, this analysis showed a rapid division frequency in all of IgG^high^ clones, while 3 IgG^−/low^ MDA-MB-231 clones showed limited divide ability, or slow growth (Figure 5I), moreover, after cryopreservation, the IgG^−/low^ clone, but not IgG^high^ clones, lost their division and growth ability. In addition, we analyzed the expression level of IgG heavy chain in MDA-MB-231 cells cultured in 3D soft fibrin gel, an ideal method to prepare CSCs [43], for 5 days. Obviously, compared to conventional cultured MDA-MB-231 cells, the MDA-MB-231 cells cultured in 3D soft fibrin gel showed overexpression level of IgG heavy chain (data not shown). This results indicated that IgG^high^ cells has higher potential of stem/progenitor cells than IgG-/low cells.

To confirm that RP215^high^ cells have tumor initiating abilities, purified RP215^high^ and RP215^−/low^ cells from MDA-MB-231 were used to perform tumorogenicity assays in NOD/SCID mice. As shown in Figure 5J and 5K, as few as 500 purified RP215^high^ cells initiated tumor formation in transplanted mice, but no or few tumor formation was found in NOD/SCID mice transplanted by the parental RP215^−/low^ cells. In addition, to further confirm the IgG link to potential of stem/progenitor cells, we have purified the 500 IgG^high^ cells and IgG^−/low^ cells from the graft tumor, which were used to perform tumorogenicity assays in NOD/SCID mice. As shown in Figure 5J, the IgG^high^ cells showed higher initiated tumor formation ability than that of IgG^−/low^ cells. We further compared the metastatic property between IgG^high^ cells and IgG^−/low^ MDA-MB-231 cells in nude mice after sub-cutaneous injection. These results showed that the IgG^high^ cells had higher metastasize capacity to the liver than IgG^−/low^ cells (Figure 5L). Expectedly, some nude mice that were injected with “IgG^high^” cells were often accompanied by ascites, pleural hemorrhage, blindness and short survival times. However, none of these phenomena were observed in mice adoptively transferred with IgG^−/low^ cells (Supplementary Figure S4). These findings suggested that the IgG^high^ cells have a much greater metastatic potential.

### RP215 recognized IgG is essential for the anchorage of cancer cells to extracellular matrix or adhere between cells, knockdown of IgG reduces proliferation, self-renewal, migration and invasion of MDA-MB-231 cells

**Figure 6:**
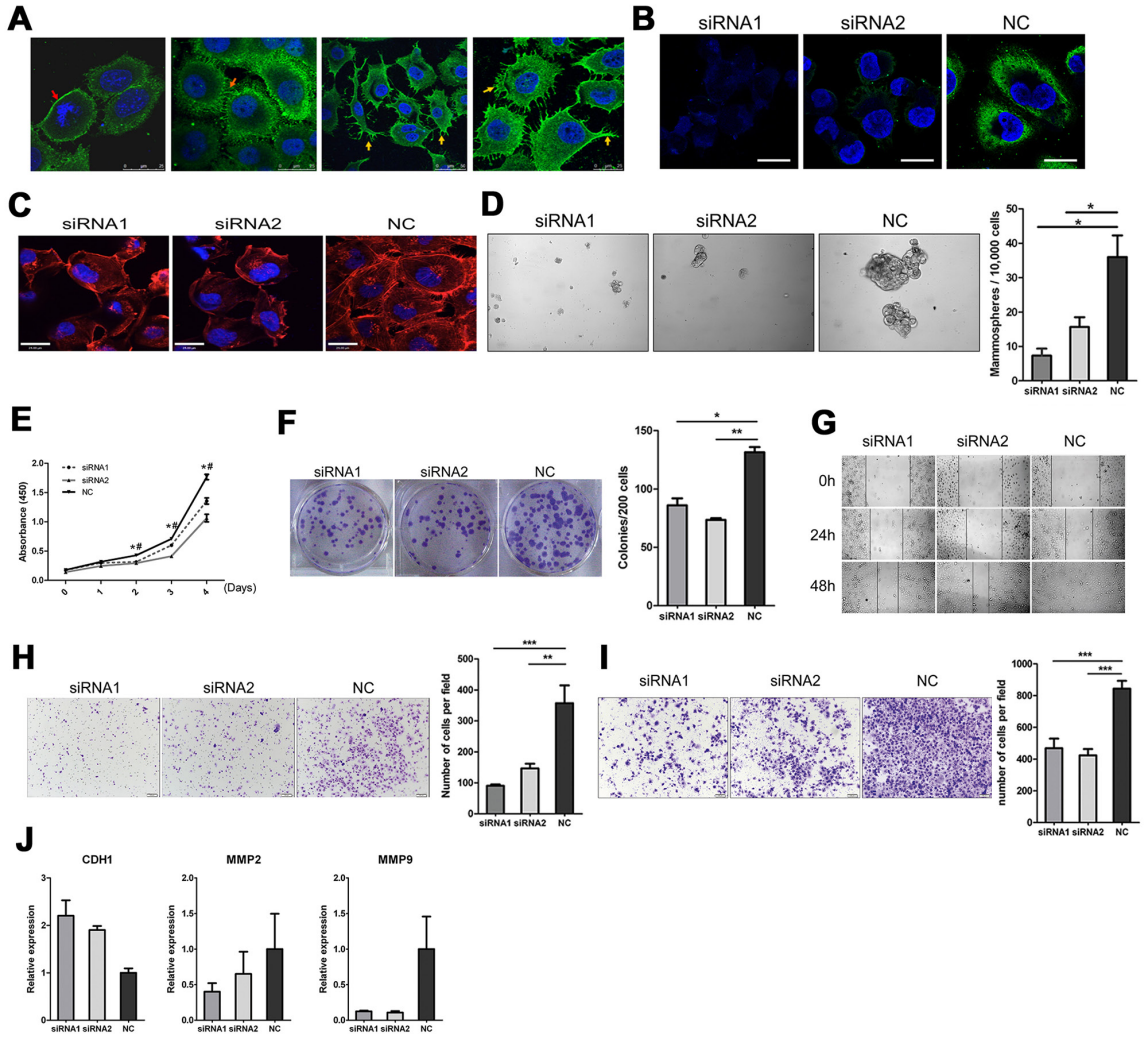
Sub-cellular localization of IgG showed on cancer cell surface, knockdown of IgG by siRNA targeting the heavy chain constant region of IgG reduces proliferation, migration, and invasion of the MDA-MB-231 cells **A.** By immunofluorescence staining, RP215-recognized IgG significantly showed on cell membrane (red arrow) filamentous junction between cancer cells (orange arrow), and pseudopodia-like structure (yellow arrow) in HT-29, MDA-MB-231 and MCF-7. Scale bars, 25 μm. **B.** IgG expression was determined after treatment with two siRNAs targeting IgG heavy chain, by Western blotting. NC as control siRNA. **C.** Morphological changes of MDA-MB-231 cells when knockdown IgG in 3D culture system (72 h) by Rhodamine-phalloidin staining for F-actin. Scale bars, 24 μm. Color: red (F-actin), blue (nuclei). **D.** Mammospheres formation after replanting of 10,000 MDA-MB-231 cells. Cell proliferation **E.** and colony formation **F.** was analyzed after treatment with IgG siRNA by CCK8 and colony formation assays. Migration ability of MDA-MB-231 cells was analyzed after treated with the IgG siRNA by wound-healing **G.** and Transwell assays **H.** Images were taken at 0, 24, and 48 h in wound heal assays and 24 h in Transwell assay. **I.** Invasion ability of MDA-MB-231 cells was analyzed after treated with the IgG siRNA on Transwell inserts coated with matrigel matrix. Images were taken at 48 h. **J.** Expression level of CDH1, MMP-2 or MMP-9 was detected after treated with the IgG siRNA by qRT-PCR. Error bars show means ± standard errors from individual experiments with three replicate assays.

We have investigated sub-cellular localization of RP215-recognized IgG in several cancer cell lines, including HT-29 (colon cancer), MDA-MB-231 and MCF-7. Immunofluorescence results show that RP215 staining mainly showed on the cell surface, but the staining degree showed considerable variations among cancer cells of the same cancer cell line. Notably, the RP215 staining on cancer cell surface displayed significantly pseudopodia-like structure, which extended to the extracellular matrix or adjacent cells (Figure 6A). We synthesized two siRNAs that targeted the constant region of the IgG heavy chain and transfer it to MDA-MB-231(Figure 6B). We found that when knockdown of IgG abolished the anchoring of cancer cells to extracellular matrix, and resulted in cell membrane rolling up (Figure 6C) in the 3D culture. Importantly, the CSC characteristics, such as self-renewal, proliferation, migration, and invasion were eliminated (Figure 6D–6I). Furthermore, less of the IgG significantly enhanced the expression of CDH1 and reduced the expression of MMP-2 and MMP-9 (Figure 6J). In addition, to test the specificity of the siRNAs, the siRNA1 and siRNA2 were mutated (the 9^th^–12^th^ base sequence were reversed, and named con-siRNA1 and con-siRNA2, respectively), and used as a control. As expected, these cell characteristics and phenotype as described above were specifically eliminated by siRNA1 and siRNA2, but not by con-siRNA1 or con-siRNA2 in MDA-MB-231 cells (Supplementary Figure S5).

## DISCUSSION

In this report, using RP215, a mAb specifically recognizing IgG heavy chain expressed in non-B cancer cells, we identified the RP215-recognized IgG, which was significantly expressed in epithelial lineage cells and was enriched in CSCs and adult stem/progenitor-like cells of the epithelial tissues. These IgG was found to be significantly associated with increased tumor initiation, migration, metastasis, and maintenance of CSCs feature, suggesting that the RP215-recognized IgG showed the function as a novel oncogene, which showed a potential for cancer therapy as a novel target of many epithelial cancers.

RP215 is a smart antibody for the recognition of epithelial cancer cell-derived IgG. In previous studies, almost all of observations about cancer cell-derived IgG at protein level was according to the finding by some commercialized anti-IgG antibody [1–12] using circulating IgG as an antigen. Those antibodies do not discriminate B cell-derived IgG from cancer cell-derived IgG. So to obtain specific antibody for the non-B-IgG is essential to findings of unique structure and function of non-B-IgG. Although it has been reported that RP215 recognizes cancer-IgG, it was questioned if RP215 may also recognize other proteins. In this study, we have further identified the specific of RP215. Our results clearly show that RP215 only recognizes a single 55 kDa IgG heavy chain in the whole cell lysate of cancer cells. This conclusion was supported by SDS-PAGE, Western blot and mass spectrometry analysis and was confirmed genetically by RNA interference of IgG. Thus, we can give a conclusion that RP215 only recognizes IgG, but not other proteins. Importantly, RP215 only recognized a portion of IgG subspecies of circulating IgG, but not the majority of circulating IgG. These results suggest that RP215 seem mainly recognize the non B cell-derived IgG, which may be used to discriminate the source of IgG between that from B cells and non B cells.

RP215-recognized IgG can serve as a novel target for therapy of most epithelial cancers, as well as which can also use for identification of epithelial cancer stem cells. RP215 have been reported to recognize many tissue origins, but not in normal cells. Moreover, RP215 displayed a great potential for cancer therapy [21, 25]. Actually, the detail expression profile of IgG recognized by RP215 still remains unclear. In this study, we first analyzed the expression profile of IgG in many epithelial, mesenchymal and lymphoid lineage of either normal or malignant cells by Immunohistochemistry. The results revealed that in the cancer tissues, RP215-recognized IgG was significantly expressed in almost all epithelial cancers, especially overexpression in a small population of epithelial CSC-like cells. Obviously, but not in either lymphoid or mesenchymal originated cancers. In normal tissues, RP215 staining did not found in almost differentiated cells, except for a small population of adult stem/progenitor cells like stem cells and squamous cells. This finding suggests that RP215-recognized IgG can serve as a novel target for therapy of almost epithelial cancers, as well as which can also use for identification of epithelial cancer stem cells. in view of the fact that RP215-recognized IgG can be found in some normal adult stem/progenitor cells like a stem cells and squamous cells, it is worth emphasizing that we have tried to determined the anti-cancer effect and safety of RP215 *in vivo*, we found that RP215 displayed a significance anti-cancer effect (data not shown), furthermore, the use of RP215 did not cause damage of trachea, breast, stomach, intestine and liver, in addition to epidermal desquamation (data not shown).

RP215-recognized IgG is involved in cancer initiation and metastasis. Cancer stem cells are cancer cells that possess characteristics associated with normal stem/progenitor cells, specifically the ability to give rise to all cell types found in a particular cancer sample, CSCs are therefore tumorigenic [44]. In this study, we have discovered that RP215-recognized IgG was overexpressed CSC-like cells such as CD44^+^/CD24^−/low^ of breast cancer cells, and the p63^+^ adult stem/progenitor cells of many stratified epithelium. Furthermore, we found that in breast cancer cell line, MDA-MB-231, IgG^high^ cancer cells displayed properties of CSCs, such as self-renewal, drug resistance, highly capacity of division, invasion, metastasis *in vitro*, and tumorigenicity in NOD/SCID mice. In contrast, knockdown of IgG heavy chain significantly reversed these phenotype described as above. More importantly, we also found that IgG heavy chain was significantly increased in the MDA-MB-231 cells cultured in 3D soft fibrin gel, an ideal method to prepare CSCs [43]. These functional studies suggest that RP215-recognized IgG may play significant roles in tumor initiation and maintenance of CSCs. In addition, in the breast carcinomas, how to understand why the RP215-recognized IgG mainly correlated with the basal-like-origin of MDA-MB-231 of BCSC that show CD44^+^/CD24^−/low^, but not with the luminal-like breast cancer cell line MCF-7 of BCSC as well as the CD133^+^ or ALDH1^+^ BCSC. Currently, we can not give an explanation. It has been considered that CD44^+^/ CD24^−/low^ cells or P63^+^ cells were mainly associated with the CSCs of basal cell origin of ductal carcinoma, however, the characteristics and markers of luminal cell-derived breast cancer CSCs remains unclear. In view of IgG expression was highly correlated with CD44^+^/CD24^−/low^ cells, we believe the IgG functions that promoting cancer genesis and metastasis are mainly association with CSCs of basal cell origin. However, it remains unclear why IgG expression was no such correlation with CSCs properties of luminal cell origin although the IgG can be also expressed in luminal cell origin of cancer cells as MCF-7.

In addition, we found that the RP215-recognized IgG was significantly located on membrane of cancer cell, especially on its pseudopodia, an essential subcellular structure for cell migration and invasion. RP215^high^ cancer cells displayed high ability of migration and metastasis. Loss of IgG abolished the anchoring of cancer cells to extracellular matrix, causing cell membrane rolling up, reducing cell migration and invasion, and inducing apoptosis. We suggest that expression of highly RP215-recognized IgG is a novel biomarker for cancer metastasis.

RP215 can be used to indicate poor prognosis and metastasis of epithelial cancers. Recently, we have reported that RP215 can be used to indicate poor prognosis and metastasis of lung adenocarcinoma [45]. In this study, our results significantly revealed that RP215 have a potential application for indicating poor prognosis and metastasis of breast cancer.

In summary, we have uncovered that RP215-recognized IgG was specifically overexpressed in many epithelial lineage cancer cells, especially enriched in epithelial CSCs. Importantly, the IgG promotes cancer adhesion, invasion and self-renewal shall be of paramount importance in order to develop novel cancer therapeutic strategy directly against the cancer-derived IgG.

## MATERIALS AND METHODS

### Tissue samples

Normal epithelial cancer and soft-tissue microarray (TMA) were purchased from ShanXi ChaoYin Biotechnology Co., Ltd., (Xi'an, China). The breast cancer TMA contained 45 pair of primary breast cancers and its metastasis cancers in lymph node (the details are available from http://www.cybrdi.cn/tissue-arrays). Paraffin-embedded samples of human breast cancer tissues were from the Department of Pathology, Beijing 301 Hospital (Beijing, China).

### Immunohistochemistry

Antigen retrieval was conducted by immersing slides in Tris-EDTA buffer (pH 9.0) at 120°C for 5 min. Sections were pre-incubated with 3% H_2_O_2_ for 10 minutes and blocked in PBS plus 10% normal goat serum for 20 min. The sections were incubated with primary antibodies including RP215 (gifted by Prof. Gregory Lee), anti-p63 (Gene Tech), anti-CD44v6 (Invitrogen, California, USA), anti-CD24 (Invitrogen) and anti-CK5/6 (Dako Cytomation) overnight at 4°C according to the manufactures' recommendations. After thorough washing, sections were exposed to Envision System horseradish peroxidase (DakoCytomation) for 20 minutes, sections were visualized with 0.05% 3, 3′-diamino-benzidine.

### SDS-PAGE and western blotting

Whole-cell extracts were prepared in cell lysis buffer (10 mM Tris-HCl, 1% Triton-X 100, 1% sodium deoxycholate, 0.1% sodium dodecyl sulfate [SDS], 0.15 M NaCl, with protease inhibitor cocktail from Roche Applied Science [Indianapolis, IN]). Proteins were resolved by 12.5% SDS–polyacrylamide gel electrophoresis (PAGE) after denaturation with heat. The objective bands were cut and subjected to trypsin digestion after stained with Coomassie brilliant blue. Following digestion, mixtures of peptide fragments were subjected to MALDI-TOF MS analysis. Western blotting analysis was performed complying with standard procedures. Goat anti-human IgG (gamma chain specific) polyclonal antibody from Sigma Chemical (St. Louis, MO), RP215 (generated by Gorge Lee group), and biotinylated elderberry bark sialyl-lectin antibody (Vector Laboratories, California) were the primary antibodies. Following incubation with IRDye 700-conjugated anti-mouse and IRDye 800-conjugated anti-goat or IRDye 800–conjugated streptavidin secondary antibodies (Rockland Immunochemicals, Gilbertsville, PA), signals was detected on an Odyssey Infrared Imager (Li-COR Biosciences, Lincoln, NE).

### Cell proliferation assay

Cell growth was determined by a CCK8 colorimetric growth assay. Briefly, cells were cultured in 96-well plates (2 × 10^3^ cells per well) in complete medium till confluence. Each day, cell growth was determined by adding 10 μl CCK8 solution in 100 μl DMEM with 10% FBS for 2 h at 37°C, and then the absorbance was measured with a 450-nm filter. This study was done in triplicate.

### Colony formation assay

Cells were seeded into each well of 6-well plates (200 cells per well). 1 week later, the cells were stained with 0.1% crystal violet to assess colony formation. Colonies containing >40 cells were counted. This study was done in triplicate.

### *In vitro* wound-healing assay

The scratch method was applied to evaluate cell migration. MDA-MB-231 cells (1 × 10^5^) were grown to confluence on 24-well plates in DMEM (1 ml per well) with 2% FBS. After 24 h, a scratch was introduced with a yellow pipette tip. Cells were further cultured for 24 h or 48 h, and then areas refilled by migrating MDA-MB-231 cells were documented. Images of four selected areas were captured and analyzed every 24 h until the wound was healed. This study was done in triplicate.

### Transwell migration and matrigel invasion assays

Transwell insert chambers with an 8 μm porous membrane (BD Biosciences, Billerica, MA) were used for the migration assay. For invasion assay, Costar transwells (Corning, Lowell, MA) coated with Matrigel (BD Biosciences) was used. Cells were added to the top chamber in serum-free medium, and the bottom chamber was filled with medium containing 10% FBS. Cells were incubated for 24 h or 48 h at 37°C in a humidified 5% CO_2_ atmosphere. Cells in the top chamber were removed by a cotton-tipped swab, and the migrated cells were stained with crystal violet. They were then visualized under a phase-contrast microscope and photographed. These studies were done in triplicate.

### Immunofluorescence staining

Cells were fixed with 4% paraformaldehyde, permeabilized with 0.1% Triton X-100/PBS for 5 min. After blocking with 2% FBS in PBS, cells were incubated with the primary antibodies against IgG, CK18, vimentin, or microtubulin for 1 h at room temperature. Secondary antibodies were goat anti-mouse or anti-rabbit labeled with Alexa 488 or TRITC (Jackson ImmunoResearch Laboratories, Inc.) for 1 h. Nuclei were counterstained with 4, 6-diamidino-2-phenylindole (Sigma). Confocal images were acquired with a TCS S2 microscope adapted to a DMIRBE inverted microscope (Leica Microsystems, Inc.).

### Mammosphere culture

Single-cell suspensions of cancer cells were suspended at a density of 10,000 cells/ml in DMEM medium containing 2% B27 (Invitrogen Ltd., Paisley, Scotland), 20 ng/ml epidermal growth factor and 10 ng/ml basic fibroblast growth factor (Peprotech, Ness-Ziona, Israel), and seeded into ultra low-attachment 24-well plates (1 ml per plate). After cultured for 1 week, the cells was collected and transferred on a collagen coated dish in cell culture medium (containing10% FBS). After approximately 48 hours, mammospheres adhered in these conditions were stained with methyl blue and counted under low magnification. This study was done in triplicate.

### Assay of clone formation and division abilities of single cells

Both of IgG^+^ and IgG^low/−^ single cancer cell was sorted into 96-well plates (1 cell per well) by FACS, and culture in complete medium, then the number of clone formation was monitored. For assay of division abilities of single cell, the single cell clones of IgG^+^ or IgG^low/−^ cell was continuously cultured and the number of cell passage was determined for 35 days.

### Quantitative RT-PCR (qRT-PCR)

qRT-PCR was carried out on 25 ng cDNA samples using platinum SYBR Green Master mix (Applied Biosystems, Carlsbad, CA). Assays were run in triplicate on the ABI Prism 7900HT system. Primers used for CDH1, MMP-2, and MMP-9 amplification were as follows: CDH1, 5′-TGCCCCCAATACCCCAGCGT-3′ (forward), 5′-TCCCTGTCCAGCTCAGCCCG-3′ (reverse); MMP-2, 5′-GACCA-TGCGGAAGCCACGCT-3′ (forward), 5′-CACCAGTGCCTGGGGCGAAG-3′ (reverse); MMP-9, 5′-GAGCCACGGCCTCCAACCAC-3′ (forward), 5′-GAG TCCAGCTTGCGGGGCAG-3′ (reverse). To ensure equal loading of cDNA into reverse-transcription reactions, 18S ribosomal mRNA was amplified using the following primers: 5′-GGAAGGGCACCACCAGGAGT-3′ (forward), 5′-TGCAGCCCCGGACATCTAAG-3′ (reverse). Data were extracted from the linear range of amplification. All quantifications were normalized to the endogenous control (18sRNA).

### siRNA transfection

The siRNAs against Ig gamma chain constant region (siRNA1 and siRNA2) and the control RNA (NC) (siRNA1: 5′-GGUGGACAAGACAGUUGAG-3′, siRNA 2: 5′-AGUGCAAGGUCUCCAACAA-3′, NC: 5′-UUCUCCGAACGUGUCACGU-3′) purchased from Shanghai GenePharma Corporation, China. Cell density was adjusted to 2 × 10^6^/350 μl. The knockdown efficiency of IgG was checked by Western blot.

### Animal model

NOD/SCID mice were used to assess the *in vivo* stem cell properties of the IgG^high^ MDA-MB-231 cells compared to the IgG^low^ MDA-MB-231 cells. BALB/c nude mice were used to evaluate both of the metastasis properties of the IgG^high^ MDA-MB-231 cells and tumor proliferation ability of MDA-MB-231 cells that treated by siRNA for IgG heavy chain. 5-week-old healthy female NOD/SCID and BALB/c nude mice were purchased from Vital River Laboratories (Beijing, China). All animals were used for *in vivo* experiments in accordance with the approved institutional guidelines of the People's Republic of China Ministry of Health and by protocols approved by the Animal Care and Use Committee of Peking University Health Science Center. The mice were allowed to acclimate for 1 week after arrival.

To the analysis of tumor initiating abilities using a few cancer cells, we have performed the tumorigenicity assay using both cultural MDA-MB-231 cells and the MDA-MB-231 cells purified from the graft tumor, respectively. The cultural MDA-MB-231 cells were stained using RP215, and IgG^+^ or IgG^low/−^ cells was sorted by FACS. 500 IgG^+^ or IgG^low/−^ cells was suspended in 50 μl of a 1:1 mix of PBS and Matrigel (BD Biosciences, Bedford, MA, USA), then were transplanted subcutaneously into the mammary fat pads of 5-week-old NOD/SCID mice (Vitalriver, Beijing, China). After 5–8 weeks, the NOD/SCID mice were sacrificed and the number of tumor formed was measured. A portion of each fat pad injected was fixed in formalin and embedded in paraffin for histological analysis. H&E staining was performed.

To analysis of tumor metastasis abilities of MDA-MB-231 cells to liver in BALB/c nude mice, 1.0 × 10^6^ cancer cells in 50 μl PBS were injected subcutaneously into the mammary fat pads. Six weeks after transplantation, the mice were sacrificed and dissected. The livers were weighed, measured, and fixed with 10% buffered formalin and embedded in paraffin for histological analysis.

### 3D cell culture

Rat-tail type I collagen was purchased from BD Biosciences. The pH of the collagen solution was adjusted to 7.4 with 1 N NaOH, cellular mixtures were made by adding MDA-MB-231 cells prepared in normal culture medium to the collagen solution; cell concentrations were adjusted to 1 × 10^6^ cells/ml. The collagen solution becomes a gel after incubation for 1 hour at 37°C [46].

### Statistical analysis

All data were analyzed with GraphPad Prism software and presented as a mean ± standard error of the mean. Statistical significance was determined by the two-tailed unpaired *t*-test with a significance level of 0.05. The Pearson correlation coefficients of IgG compared to that of CD44v6 was analyzed via Matlab software (Mathworks).

## SUPPLEMENTARY FIGURES AND TABLE


