# Supplementary material for: Aberrant high expression of immunoglobulin G in epithelial stem/progenitor-like cells contributes to tumor initiation and metastasis
Source: Oncotarget. 2015 Oct 12;6(37):40081–94. doi: 10.18632/oncotarget.5542 (PMC4741881; doi:10.18632/oncotarget.5542)
Supplement: Supplementary file 1 [file oncotarget-06-40081-s001.pdf]

SUPPLEMENTARY FIGURES AND TABLE

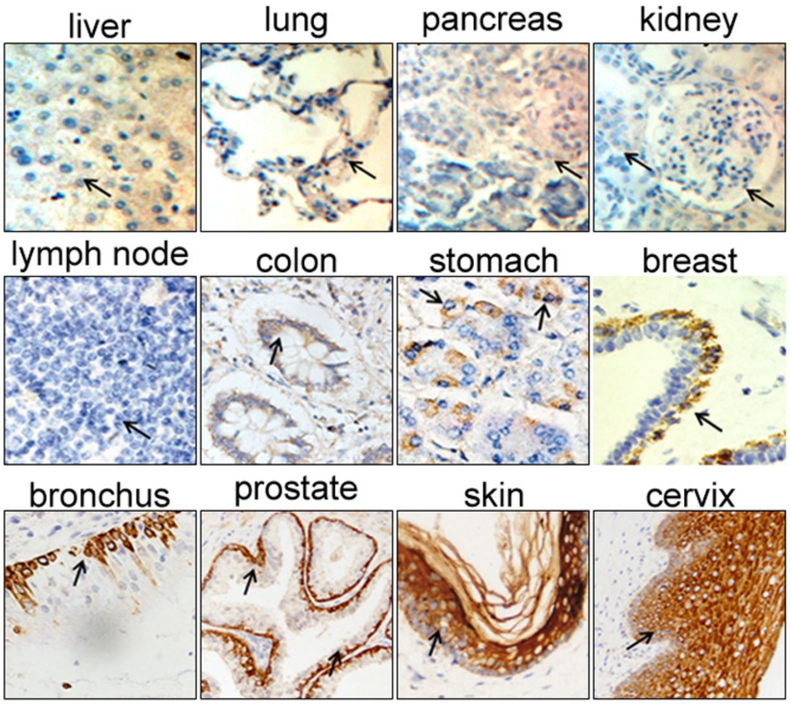

Supplementary Figure S1: Expression profile of RP215-recognized IgG in normal tissues. No RP215 recognized IgG stanining showed in normal liver, lung, pancreas, kidney, lymph node, a few of cells staining in colon, stomach, bile duct. Strong staining in some basal layer/myoepithelial cells of breast glands, prostatic glands as well as in squamous cells of skin, esophagus and cervix.

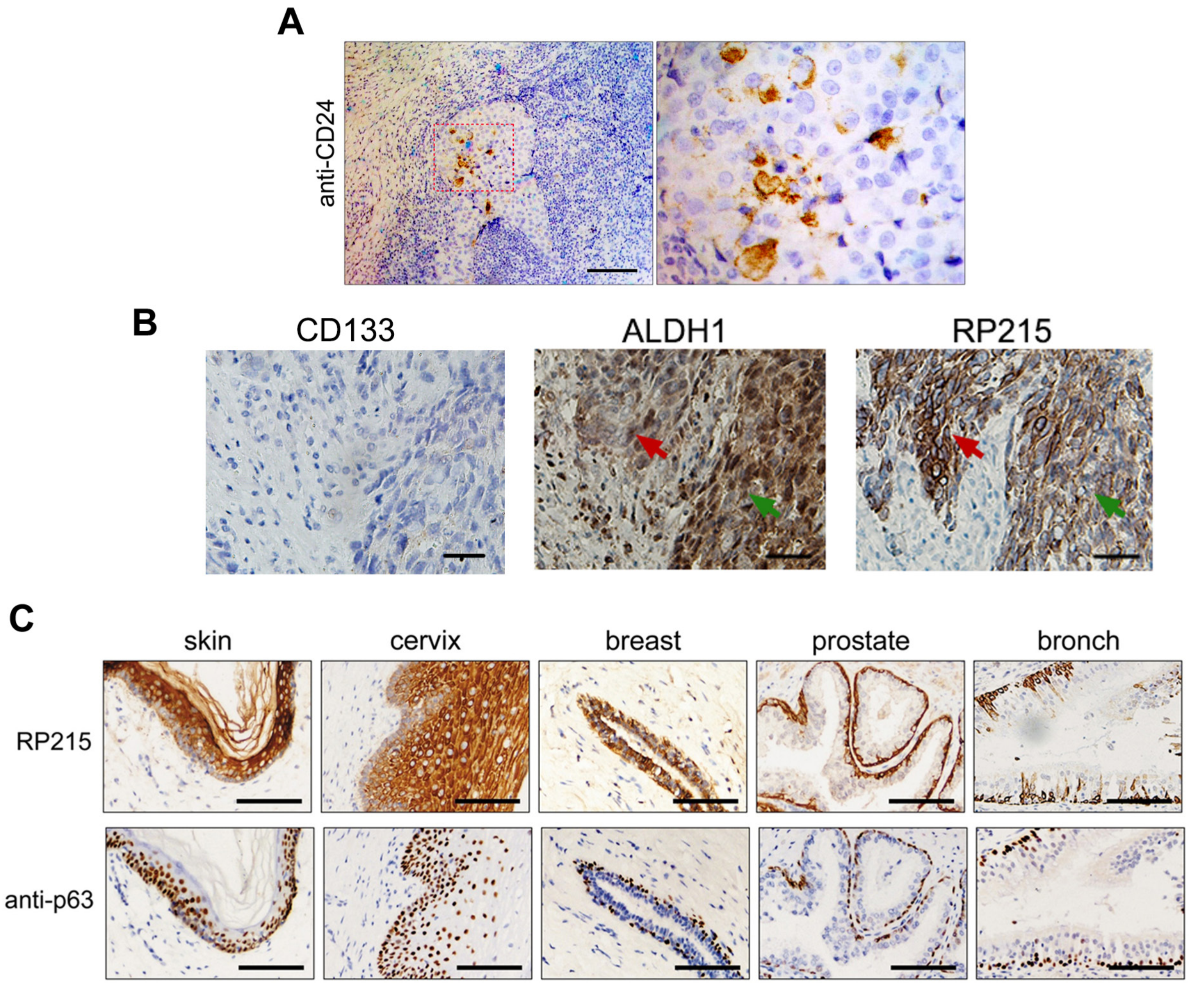

Supplementary Figure S2: A. $CD24^+$ cells were mainly located in the cancer nest. Scale bar: 100 µm. **B.** No significant correlation was found between either IgG^{high} cells and CD133, or IgG^{high} cells and ALDH1. Negative CD133 staining was shown. Some cells which are strong positive RP215 staining show weak or negative ALDH1 staining (Red arrow), while the weak RP215 staining cells show strong ALDH1 staining (Green arrow). **C.** The IgG staining was high revealed in some normal epithelial cells with stem/progenitor properties, which is similar to that for p63, an adult stem/progenitor marker. Scale bars, 100 µm.

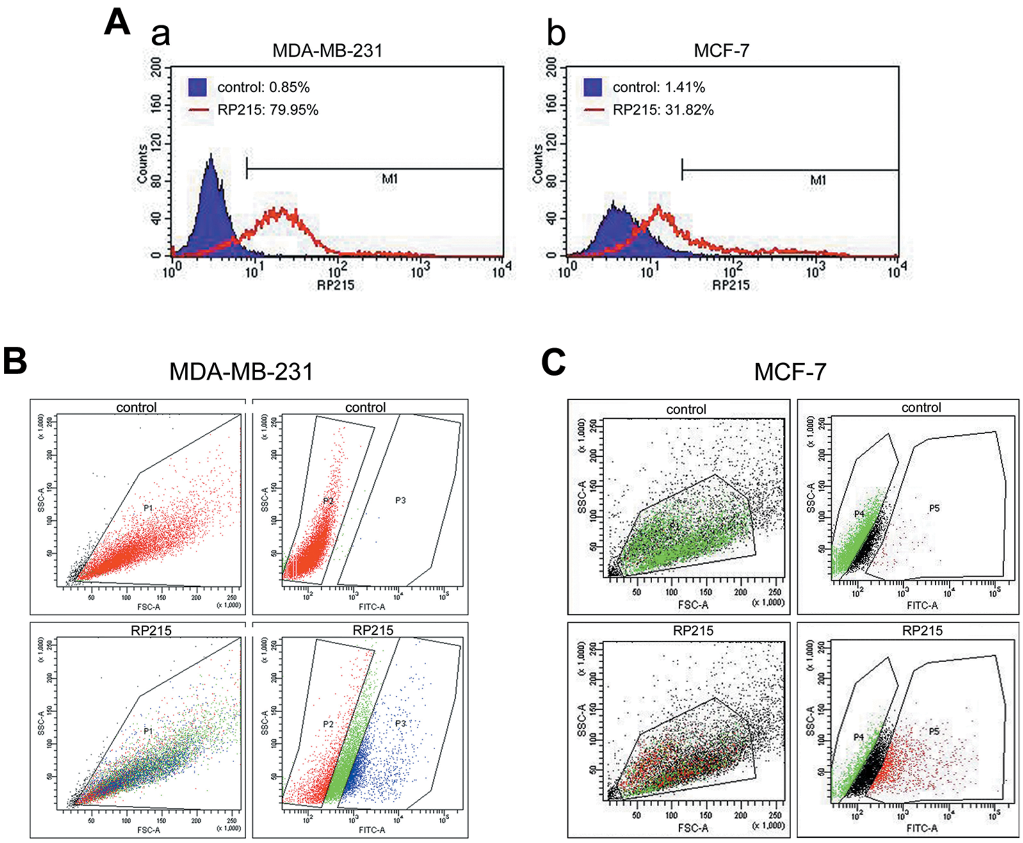

Supplementary Figure S3: A. The percentage of RP215-recognized cells in MDA-MB-231 (a) was higher than in MCF-7 (b) by FACS. **B.** and **C.** RP215^{high} and RP215$^{-/low}$ MDA-MB-231 cells or MCF-7 cells were sorted by FACS using RP215. Mouse IgG was used as isotype control. P1: live cells; P2: RP215$^{-/low}$ MDA-MB-231 cells; P3: RP215^{high} MDA-MB-231 cells; P4: RP215$^{-/low}$ MCF-7 cells; P5: RP215^{high} MCF-7 cells. The P2, P4 (as RP215$^{-/low}$) and P3, P5 cells (as RP215^{high}) were selected respectively.

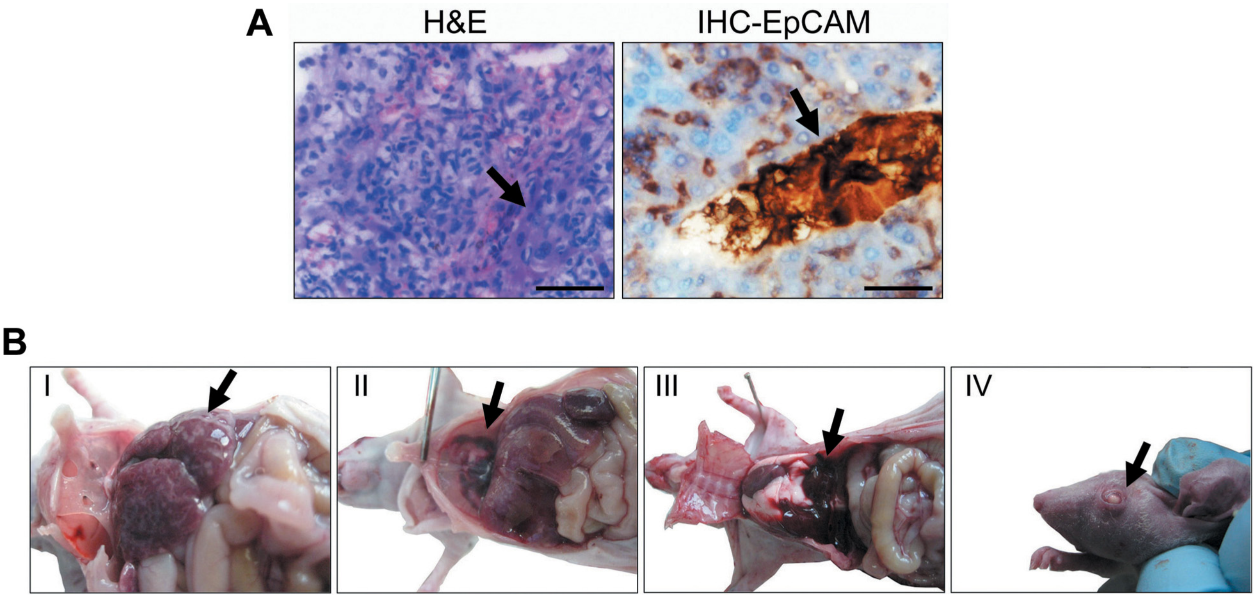

Supplementary Figure S4: A. The RP215^{high} MDA-MB-231 cells revealed strong metastatic ability *in vivo*. Hematoxylin & eosin staining showed metastases of human breast cancer to liver of nude mice, and using the specific antibody against human EpCAM antibody, showed metastases of human breast cancer in the livers of nude mice by immunohistochemistry (arrows). Scale bars, 50 μm. **B.** Injection of RP215^{high} MDA-MB-231 cells into nude mice resulted in apparent ascites and hemothorax, even blindness (arrows).

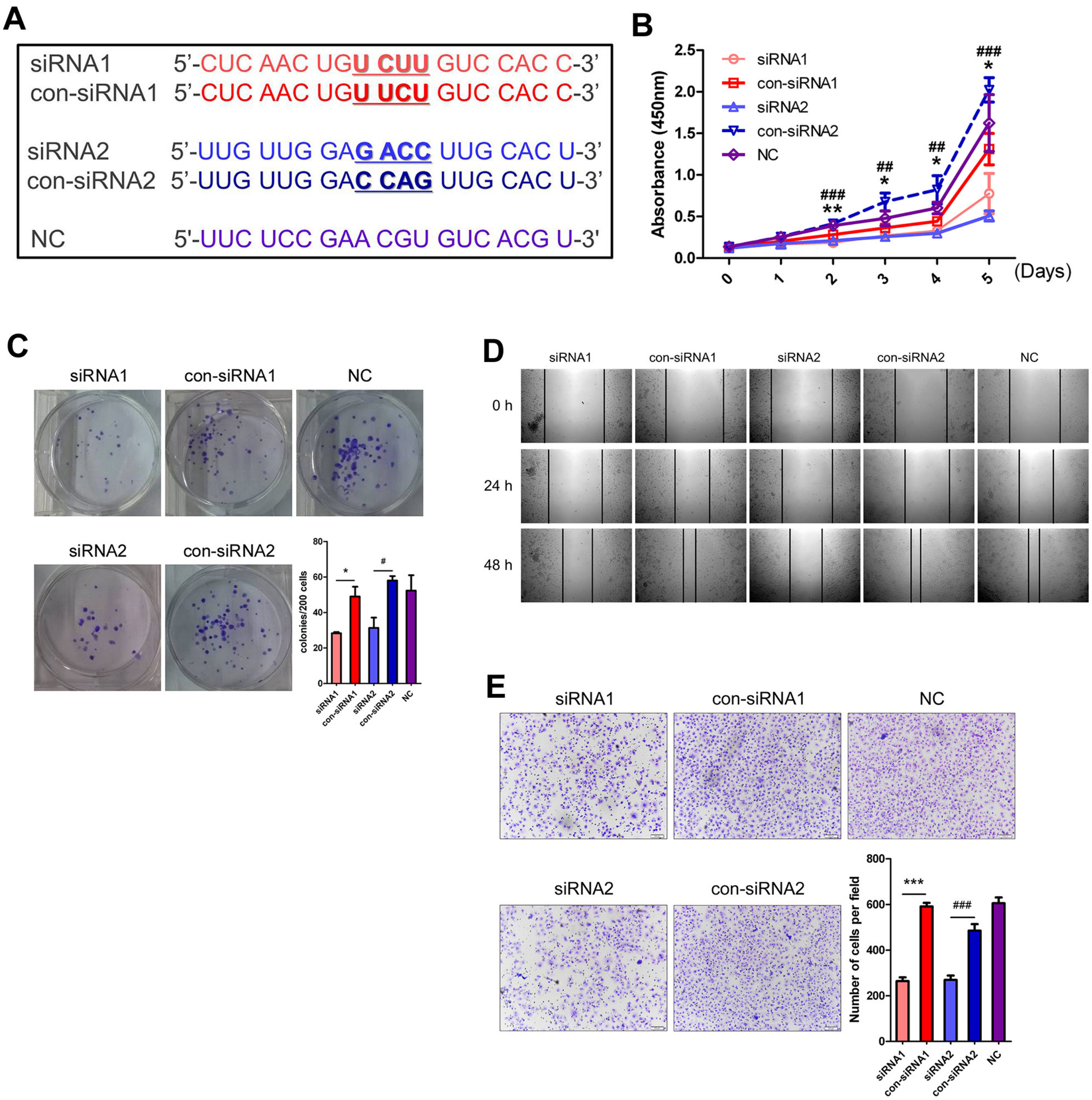

Supplementary Figure S5: Cell proliferation, colony formation and migration ability of MDA-MB-231 was analyzed after treatment with siRNA1, con-siRNA1, siRNA2, con-siRNA2. A. The sequences list of siRNA1, con-siRNA1, siRNA2, con-siRNA2 and NC. **B.** Cell proliferation was analyzed by CCK8; **C.** Colony formation assay; **D.** and **E.** Migration ability was analyzed by wound-healing and Transwell assays. Images were taken at 0, 24, and 48 h in wound heal assays and 36 h in Transwell assay. *: siRNA1 compared con-siRNA1, #: siRNA2 compared con-siRNA2, */# $P < 0.05$; **/## $P < 0.01$; ***/### $P < 0.001$ by the Student *t*-test.

Supplementary Table S1: Using a human soft-tissue microarray (TMA), detects the expression of RP215 in mesenchymal tumor tissues

Pathology diagnosis & type	Cases	Positive cases	Comments for positive cases
Fibrolipoma (B)	1	0	
Lipoma (B)	1	0	
Fibroma (B)	1	0	
Stromal tumor (B)	2	0	
Angioleiomyoma (B)	1	0	
Hemagioma (B)	2	0	
Leiomyoma (B)	4	0	
Chondrosarcoma (M)	4	0	
Fibrosarcoma (M)	8	0	
Pleomorphic rhabdomyosarcoma (M)	2	0	
Alveolar rhabdomyosarcoma (M)	2	0	
Synovial sarcoma (M)	2	2	positive staining only in epithelial-like cells
Epithelioid sarcoma (M)	1	1	positive staining only in epithelial-like cells
Leiomyosarcoma (M)	6	0	
Malignant stromal tumor (M)	3	0	
Mesothelioma (M)	1	1	positive staining only in epithelial-like cells
Hemangiopericytosarcoma (M)	1	0	
Malignant mesenchymoma (M)	1	0	
Liposarcoma (M)	2	0	
Malignant fibrous histiocytoma (M)	3	0	

B: benign; M: malignant.
